# The effect of menaquinone-7 supplementation on dp-ucMGP, PIVKAII, inflammatory markers, and body composition in type 2 diabetes patients: a randomized clinical trial

**DOI:** 10.1038/s41387-022-00192-5

**Published:** 2022-04-01

**Authors:** Nahid Karamzad, Esmaeil Faraji, Shaghayegh Adeli, Mark J. M. Sullman, Bahram Pourghassem Gargari

**Affiliations:** 1 Department of Biochemistry and Diet Therapy, Faculty of Nutrition and Food Sciences, Tabriz University of Medical Sciences, Tabriz, Iran; 2 Student Research Committee, Tabriz University of Medical Sciences, Tabriz, Iran; 3 Nutrition Research Center, Faculty of Nutrition and Food Sciences, Tabriz University of Medical Sciences, Tabriz, Iran; 4 Endocrine Research Center, Tabriz University of Medical Sciences, Tabriz, Iran; 5 Department of Life and Health Sciences, University of Nicosia, Nicosia, Cyprus; 6 Department of Social Sciences, University of Nicosia, Nicosia, Cyprus

**Keywords:** Type 2 diabetes, Nutrition disorders

## Abstract

**Background:**

Type 2 diabetes mellitus (T2DM) is a common disorder that is characterized by chronic hyperglycemia and chronic inflammation, which also have a reinforcing effect on each other. The present research studied the effects of menaquinone (MK-7) supplementation on serum dp-ucMGP (dephospho uncarboxylated Matrix Gla Protein), PIVKAII (Prothrombin Induced by Vitamin K Absence), inflammatory markers and body composition indices in type 2 diabetes mellitus (T2DM) patients.

**Methods:**

This 12-week double-blind placebo-controlled randomized clinical trial allocated 60 T2DM patients equally into a MK-7 (200 mcg/day) group or a placebo group. All patients also received dietary advice at the beginning of study and their dietary intakes were checked using a 3-day food record. The body composition of each patient was also measured and their vitamin K status was assessed using the ELISA method to measure serum dp-ucMGP and PIVKAII. In addition, inflammatory status indices were also measured, including hsCRP (high-sensitivity C-reactive protein), IL-6 (interleukin-6) and TNF-α (tumor necrosis factor alpha). All measurements were made both before and after the intervention period.

**Results:**

In total 45 patients completed the trial (MK-7 group = 23 and placebo group = 22). The calorie and macronutrient intake of the two groups were similar pre and post intervention. There were statistically significant increases in dietary vitamin K intake for both groups over the course of the study (*p* < 0.05), but the intergroup differences were not significant. The body composition indices (i.e., body fat percentage, fat mass, fat free mass, muscle mass, bone mass and total body water) were not significantly different between groups or across the trial. The serum levels of the vitamin K markers, PIVKAII and dp-ucMGP, decreased significantly in the MK-7 group over the course of the study (*p* < 0.05), but there was no decrease in the placebo group. However, after adjusting for the baseline levels and changes in vitamin K intake, the between group differences were only significant for PIVKAII (*p* < 0.05). Following the intervention, the serum levels of the inflammatory markers (hsCRP, IL-6, and TNF-α) were significantly lower in the MK-7 group (*p* < 0.05), but not in the placebo group. However, the between group differences in the inflammatory markers were not statistically significant.

**Conclusions:**

Although further studies are needed, it appears that MK-7 supplementation can be effective in improving PIVKAII levels, but not for improving dp-ucMGP, inflammatory status or the body composition indices of T2DM patients.

**Trial registration number:**

This study was prospectively registered at the Iranian Registry of Clinical Trials on the 20th of May 2019 (ID: IRCT20100123003140N22).

## Introduction

Type 2 diabetes mellitus (T2DM) is a complex multifactorial disease that is typified by insulin resistance and chronic hyperglycemia [1]. T2DM has a relatively high prevalence in Iran and from 1990 to 2017 its prevalence increased by 25% [2]. According to an increasing body of research, inflammation exists in DM patients, as shown by the increased levels of IL-6 (interleukin-6), hsCRP (high-sensitivity C-reactive protein), and TNF-α (tumor necrosis factor alpha) [3–6]. Inflammation is an important factor in insulin signaling and the pathogenesis of diabetes [7, 8]. In addition, inflammation and chronic hyperglycemia have a reinforcing effect on each other [9]. Therefore, the purpose of many dietary supplements and medications, which are used for treating DM patients, is to reduce the level of inflammation [10, 11], which can lead to a reduction in DM complications, including hyperglycemia, insulin resistance, dyslipidemia, as well as microvascular and macrovascular complications [12, 13]. Diabetes has also been recognized as one of the risk factors for vitamin K deficiency [14]. Phylloquinone (vitamin K1), menaquinone (MK or vitamin K2) and menadione (synthetic form) are different forms of vitamin K [15]. MK-4 and MK-7 are the most common forms of menaquinone, but MK-7 has a longer half-life, better bioavailability and uptake [16, 17]. Vitamin K supplementation can lead to changes in vitamin K levels, but there is no gold standard for measuring it [18]. The measurement of functional markers, like non-carboxylate vitamin K-dependent proteins, such as PIVKAII (Prothrombin Induced by Vitamin K Absence) and dp-ucMGP (dephospho uncarboxylated Matrix Gla Protein), have been utilized as indirect indicators of vitamin K status [18, 19].

There have been a limited number of studies on diabetic animals and pre-diabetic patients, which have found supplementation with vitamin K to be beneficial for diabetes and its associated complications [20]. More specifically, only a single clinical trial has examined the effect of MK-7 supplementation on patients with T2DM, although the aim of that study was different from our own [21]. The present study investigated the effect that MK-7 supplementation had on serum dp-ucMGP, PIVKAII, inflammatory markers (IL-6, hsCRP, TNF-α) and body composition (bone mass, muscle mass, body fat percentage, fat mass, fat free mass, and total body water) in T2DM patients.

## Methods

### Participants

This research was undertaken with 60 T2DM patients from August to December 2019, in Tabriz, Iran. Patients were recruited using printed advertisements and clinical referrals. All volunteers were firstly screened by phone and then in a face-to-face meeting. The inclusion criteria were: willingness to participate, aged 20–55 years, BMI 27–35 kg/m^2^ and having anti T2DM therapy (i.e., anti-diabetic drugs, but not insulin) for 6 months, or more, before the study. The exclusion criteria included those who were: current smokers; pregnant; lactating; undergoing menopause; undergoing hormone therapy; using contraceptives containing vitamin K; diagnosed with polycystic ovary syndrome; the presence of diseases associated with cardiac dysfunction or vascular calcification; using insulin; using corticosteroids or anticoagulant drugs (e.g., warfarin or coumarin); on a specific diet; consuming any dietary supplements or drugs to reduce weight in the last 3 months. In addition, potential participants were excluded if they had a family history of bone disease, rheumatoid arthritis, thyroid problems, parathyroid problems, liver disease, kidney disease, intestinal disease, malignancies, infectious or inflammatory diseases or who recently had undergone surgery.

This study is part of a larger project which has also studied the effects of MK-7 supplementation on glycemic status, anthropometric indices and lipid profile in patients with T2DM [22]. The project was registered at the Iranian Registry of Clinical Trials (IRCT20100123003140N22), after being approved by the ethics committee of the Tabriz University of Medical Sciences, Tabriz, Iran (Ethics code: IR.TBZMED.REC.1398.123).

### Study design and procedure

In this double-blind placebo-controlled randomized clinical trial, an allocation ratio of 1:1 was used. A blinded assistant, who was not involved in any other aspect of the study, used RAS (Random Allocation Software) to randomly assign participants into the two experimental groups (1:1). The sequence for randomization was generated using the balanced block randomization method. All researchers and participants were blind to participant group membership. The MK-7 and placebo capsules, containers and labels were exactly the same, with the only difference being a tri-digit code printed on the labels by our assistant to identify them. The intervention group were given 200 mcg/day of MK-7, while the placebo group were given capsules containing 100 mg of cornstarch. Participants were asked to consume the capsules with their main meal over a 12-week period. In addition, dietary advice was provided to all patients at the beginning of the study, which can be found in Table 1 of the Supplementary Appendix [23].

**Table 1:** Baseline characteristics of the study population.

Variable	Placebo group (*n* = 22)	MK-7 group (*n* = 23)	*p*
Age (year)^a^	46.95 (5.25)	45.35 (6.25)	0.35^d^
Sex^b^			
Male	15 (62.8%)	16 (69.6%)	0.92^f^
Female	7 (31.8%)	7 (30.4%)	
Education level^b^			
<diploma	10 (45.5%)	9 (39.1%)	0.91^f^
Diploma and Associate	6 (27.3%)	7 (30.4%)	
≥Bachelor	6 (27.3%)	7 (30.4%)	
Marital status^b^			
Single	1 (4.5%)	1 (4.3%)	1^g^
Married	21 (95.5%)	22 (95.7%)	
Occupation^b^			
Housewife	13 (59.1)	15 (65.2%)	0.75^g^
Employed	5 (22.7%)	6 (26.1%)	
Retired	4 (18.2%)	2 (8.7%)	
Duration of diabetes (year)^c^	4 (2)	3 (3)	0.34^e^
Drug history^b^			
Only anti diabetic drugs	3 (13.6%)	8 (34.8%)	0.17^g^
Anti-hyperlipidemia drugs	10 (45.5%)	8 (34.8%)	
Anti-hypertension drugs	3 (13.6%)	3 (13%)	
Both anti-hyperlipidemia and anti-hypertension drugs	6 (27.3%)	2 (8.7%)	
Other drugs	0 (0%)	2 (8.7%)	

^a^Mean (SD).

^b^Number (%).

^c^Median (25th and 75th percentiles).

^d^Based on an independent-samples *t*-test.

^e^Based on a Mann–Whitney *U*-test.

^f^Based on a Pearson’s Chi-Square test.

^g^Based on a Fishers exact test.

All eligible volunteers were interviewed by an endocrinologist to confirm their T2DM diagnosis. Following a full explanation of the study’s procedures, informed consent was obtained in writing and a demographic questionnaire was completed. The following day, after fasting overnight (for 12-h), blood was taken to measure biochemical parameters and body composition measures were also made. Furthermore, after explaining how to complete it, participants were also asked to fill in a 3-day food record. Aside from the demographics questionnaire, all other assessments were undertaken both at baseline and after completion of the study, 12 weeks later. In addition, participants were also asked to return to the clinic every 3 weeks, over the 12-week duration of the study, in order to follow them up, check for side effects (there were none) and to deliver the MK-7/placebo supplements.

### Dietary intake and body composition

The dietary intake of participants was assessed at baseline and after 12 weeks, using their 3-day food records (one weekend and two workdays). Their intake average across the 3 days was measured using *Nutritionist IV* software (First Databank, San Bruno, CA, USA), which was modified for Iranian foods. A Tanita MC-780 S MA (Amsterdam, the Netherlands) was used to evaluate body composition pre and post intervention.

### Biochemical measurements

Fasting blood samples were taken for serum analysis at baseline and after 12 weeks. Each 7 mL sample of blood was centrifuged at 3000 rpm for 5 min and then stored at −80 °C. After the supplementation period, the vitamin K status markers (dp-ucMGP and PIVKAII) were measured, in accordance with manufacturer’s instructions, using the enzyme-linked immunosorbent assay (ELISA) method and Shanghaicrystal Day Biotech Co. kits. Serum hsCRP, IL-6, and TNF-α levels were measured to evaluate each participants’ inflammatory status. IL-6 and TNF-α were measured using Haznghou Eastbiopharm Co. kits and the ELISA method. Finally, high-sensitivity C-reactive protein (hs-CRP) was measured using immunoturbidimetric assay (BioSystems S.A. Spain).

### Sample size and statistical analysis

The minimum sample size of 23 per group, which provided 80% power and a 5% risk of type 1 error, was selected after reviewing a similar recently published study [24]. However, a final sample size of 30 per group was selected, in order to accommodate a sample attrition rate of 30% over the duration of the study. The Kolmogorov Smirnov statistic was used to test the normality of the quantitative variables. The normally distributed variables were reported as means (±standard deviation), while those that were not normally distributed were reported as medians (25th and 75th percentiles). Categorical variables were presented as numbers and percentages. At baseline, differences between the two groups were tested using independent sample *t*-tests or Mann–Whitney *U*-tests for continuous variables, while Chi-squared or Fishers exact tests were used for the categorical variables. Within-group changes were tested using paired samples *t*-test (normally distributed) or Wilcoxon signed-rank tests (non-normally distributed). At the end of the study, continuous variables were compared between groups (i.e., intervention and placebo) using an analysis of covariance (ANCOVA) (normally distributed) or Quantile Regression (non-normally distributed), which were adjusted for baseline values and potential confounding factors. SPSS software Version 19 (SPSS Inc., IL, Chicago, USA) and Stata Version 14 (StataCorp, College Station, TX, USA) were used to conduct all statistical tests, with significance set at *p* < 0.05.

## Results

**Fig. 1:**
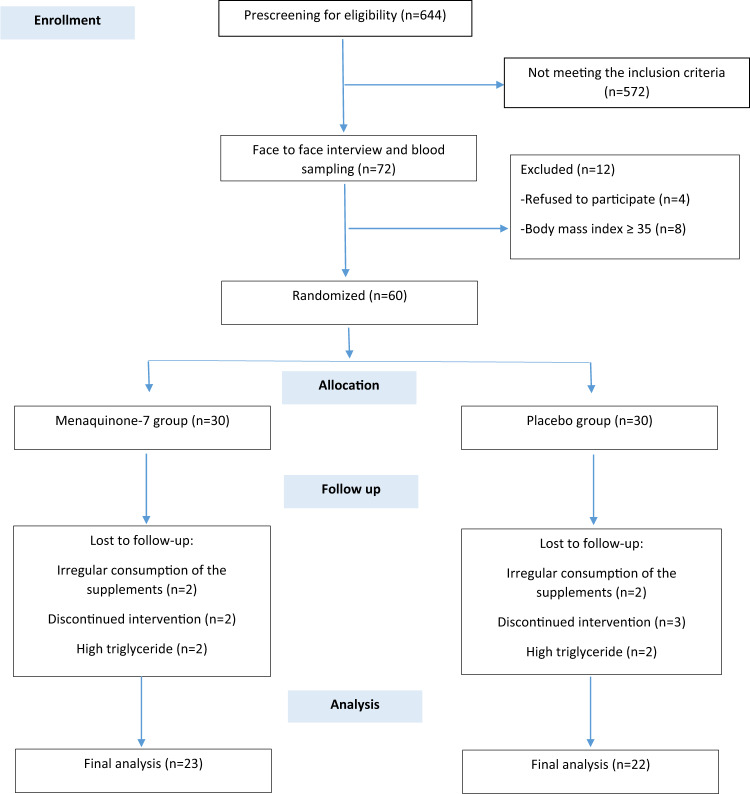
Flow-diagram of the patient selection process.

As shown in Fig. 1, 644 patients were spoken to by telephone to identify which met the inclusion criteria. After face-to-face meetings with 72 of them, 12 patients were excluded because they had a BMI > 35 or they decided not to participate. The study was started with sixty T2DM patients, but by the end of the study seven had been lost from the MK-7 group and eight from the placebo group. Therefore, the study was completed by 45 patients (MK-7 group *n* = 23; placebo group *n* = 22). The two groups were homogeneous for baseline characteristics and drug history (Table 1). The majority of the patients were married and had a lower than diploma level of education. The mean (SD) age of the patients was 45.35 (6.25) in the MK-7 group and 46.95 (5.25) years old in the placebo group.

### Dietary intake and body composition

The calorie and macronutrient intake of the two groups were similar at baseline (*p* > 0.05). Following the completion of the intervention, there were no significant changes found in either group (*p* > 0.05), and neither were there significant between groups differences (adjusted for baseline values) (*p* > 0.05) (Table 2). Although dietary vitamin K intake significantly increased for both the MK-7 (MD: 63.38; *p*: 0.001) and placebo (MD: 47.73; *p*: 0.02) groups, the between group differences were not significant before (MD: −35.34; *p*: 0.09) or after the intervention (MD: −4.03; *p*: 0.86).

**Table 2:** Comparison of the dietary intake between study groups at baseline and the end of the intervention.

Variable	Placebo group (*n* = 23)	MK-7 group (*n* = 22)	MD (95% CI), *p*
Energy (Kcal/day)			
Before	2466.31 (1105.53)	2256.36 (666.22)	209.95 (−335.96, 755.86), 0.44^b^
After	2526.49 (1087.02)	2327.57 (818.62)	148.15 (−424.37, 720.67), 0.60^a^
MD (95% CI), *p*^c^	60.17 (−503.23, 623.58), 0.82	71.20 (−379.92, 522.33), 0.74	
Fat (g)			
Before	59.67 (26.78)	57.73 (23.53)	1.93 (−13.20, 17.07), 0.79^b^
After	65.38 (28.77)	66.88 (24.33)	−1.69 (−17.82, 14.43), 0.83^a^
MD (95% CI), *p*^c^	5.70 (−9.13, 20.54), 0.43	9.14 (−6.43, 24.72), 0.23	
Protein (g)			
Before	95.63 (46.80)	84.68 (26.32)	10.94 (−11.75, 33.64), 0.33^b^
After	100.63 (45.50)	90.65 (32.45)	8.12 (−15.79, 32.03), 0.49^a^
MD (95% CI), *p*^c^	4.99 (−20.33, 30.33), 0.68	5.96 (−12.13, 24.06), 0.50	
Carbohydrate (g)			
Before	395.19 (206.86)	357.56 (108.66)	37.62 (−61.08, 136.33), 0.44^b^
After	371.21 (155.33)	351.75 (143.75)	11.83 (−77.54, 101.21), 0.79^a^
MD (95% CI), *p*^c^	−23.98 (−123.81, 75.85), 0.62	−5.81 (−76.36, 64.73), 0.86	
Vitamin K (mcg/day)			
Before	91.86 (58.98)	127.20 (79.24)	−35.34 (−77.48, 6.80), 0.09^b^
After	155.24 (86.19)	174.93 (78.26)	−4.03 (−51.91, 43.85), 0.86^a^
MD (95% CI), *p*^c^	63.38 (28.72, 98.03), 0.001	47.73 (7.91,87.54), 0.02	

Mean (standard deviation) and mean difference (95% CI) are presented for normally distributed data.

*MD* mean difference; *CI* confidence interval.

^a^*p* based on an analysis of covariance (ANCOVA), adjusted for baseline values.

^b^*p* based on an independent-samples *t*-test.

^c^*p* based on a paired samples *t*-test.

Baseline body composition indices, including: body fat percent (MD: 0.95; *p*: 0.56), fat mass (MD: 0.97; *p*: 0.50), fat free mass (MD: −0.55; p: 0.86), muscle mass (MD: −0.53; *p*: 0.86), bone mass (MD: 0; *p*: 0.99) and total body water (MD: −0.84; *p*: 0.50) did not differ significantly between the two groups (Table 3). Additionally, the within group and between group changes were not statistically different for these parameters. (Table 3).

**Table 3:** Comparison of body composition between study groups at baseline and at the end of the intervention.

Variable	Placebo group (*n* = 22)	MK-7 group (*n* = 23)	MD (95% CI), *p*
Body fat percent (%)			
Before	26.82 (5.38)	25.87 (5.42)	0.95 (−2.33, 4.24), 0.56^a^
After	26.92 (5.56)	26.47 (4.31)	−0.39 (−1.50, 0.71), 0.47^f^
MD (95% CI), *p*^b^	0.09 (−0.56, 0.75), 0.76	0.60 (−0.38, 1.58), 0.22	
Body fat mass (kg)			
Before	22.83 (5.04)	21.85 (4.68)	0.97 (−1.98, 3.93), 0.50^a^
After	23.12 (5.25)	22.62 (4.09)	−0.43 (−1.45, 0.59), 0.40^f^
MD (95% CI), *p*^b^	0.29 (−0.33, 91), 0.34	0.76 (−0.07, 1.61), 0.07	
Body fat free mass (kg)			
Before	62.71 (10.77)	63.27 (10.76)	−0.55 (−7.11, 6.00), 0.86^a^
After	63.19 (10.80)	63.44 (10.56)	0.27 (−0.72, 1.26), 0.58^f^
MD (95% CI), *p*^b^	0.47 (−0.07, 1.03), 0.08	0.16 (−0.64, 0.98), 0.67	
Body muscle mass (kg)			
Before	59.60 (10.24)	60.13 (10.26)	−0.53 (−6.77, 5.71), 0.86^a^
After	60.02 (10.28)	60.29 (10.07)	0.23 (−0.72, 1.19), 0.62^f^
MD (95% CI), *p*^b^	0.42 (−0.12, 0.97), 0.12	0.15 (−0.62, 0.93), 0.68	
Body bone mass (kg)			
Before	3.30 (2.90, 3.50)	3.30 (2.77, 3.52)	0.00, 0.99^c^
After	3.20 (2.80, 3.45)	3.30 (2.60, 3.50)	0 (−0.07, 0.07), 1^e^
MD, *p*^d^	−0.10, 0.56	0.00, 0.52	
Total body water (%)			
Before	52.74 (4.09)	53.58 (4.29)	−0.84 (−3.40, 1.71), 0.50^a^
After	52.24 (4.45)	53.05 (3.46)	−0.07 (−1.15, 0.99), 0.88^f^
MD (95% CI), *p*^b^	−0.50 (−1.21, 0.21), 0.16	−0.53 (−1.39, 0.32), 0.21	

Mean (SD) and mean difference (95% CI) are presented for normally distributed data; Median (25th and 75th percentiles), median difference and coefficient (95%CI) are presented for data not normally distributed.

^a^*p* based on an independent-samples *t*-test.

^b^*p* based on a paired samples *t*-test.

^c^*p* based on a Mann-Whitney *U*-test.

^d^*p* based on a Wilcoxon signed-ranked test.

^e^*p* based on quantile regression, adjusted for baseline values, and changes of dietary vitamin K intake.

^f^*p* based on an ANCOVA adjusted for baseline values and changes of dietary vitamin K intake.

### Vitamin K status and inflammatory markers

Baseline serum PIVKAII (MD: −3.90; *p*: 0.23) and dp-ucMGP (MD: −10.0; *p*: 0.36) did not differ significantly between the two groups. After 12 weeks supplementation, PIVKAII (MD: −8.70; *p*: 0.01) and dp-ucMGP (MD: −0.30; *p*: 0.00) levels had decreased significantly in the MK-7 group, but not in the placebo group. However, after adjusting for baseline levels and changes in vitamin K intake, only PIVKAII remained significantly lower in the MK-7 group than in the placebo group (MD: −10.50; *p*: 0.04) (Table 4).

**Table 4:** Comparison of vitamin K status and inflammatory markers between study groups at baseline and at the end of the intervention.

Variable	Placebo group (*n* = 22)	MK-7 group (*n* = 23)	MD, *p*
dp-ucMGP (ng/mL)			
Before	0.70 (0.30, 2.10)	0.80 (0.30, 11.60)	−0.10, 0.36^a^
After	0.80 (0.20, 2.57)	0.50 (0.20, 7.50)	−0.30 (−1.18, 0.57), 0.48^c^
MD*, p*^*b*^	0.10, 0.43	−0.30, 0.00	
PIVKAII (AU/mL)			
Before	14.40 (9.20, 32.35)	18.30 (10.10, 162.20)	−3.90, 0.23^a^
After	18.30 (9.27, 60.15)	9.60 (6.60, 150)	−10.50 (−20.48, −0.51), 0.04^c^
MD, *p*^*b*^	3.90, 0.11	−8.70, 0.01	
hsCRP (mg/dL)			
Before	2.16 (0.98, 5.48)	1.89 (1.41, 4.50)	0.27, 0.77^a^
After	2.03 (1.18, 3.56)	1.30 (0.48, 3.52)	−0.39 (−2.23, 1.43), 0.66^c^
MD*, p*^*b*^	−0.13, 0.48	−0.59, 0.02	
TNF-α(ng/L)			
Before	42.55 (26.65, 98.07)	121 (26.10, 547.70)	−78.45, 0.25^a^
After	40.25 (26.55, 279.42)	55.80 (15.80, 461)	−12.39 (−79.05, 54.26), 0.70^c^
MD, *p*^*b*^	−2.3, 0.37	−65.2, 0.04	
IL-6 (ng/L)			
Before	140.80 (67.19, 222.42)	170.80 (106.10, 1006)	−30, 0.32^a^
After	140.55 (107.15, 186.85)	127 (91, 884)	−14.91 (−42.87, 13.04), 0.28^c^
MD, *p*^*b*^	−0.25, 0.78	−43.8, 0.02	

Median (25th and 75th percentiles), median difference and coefficient (95%CI) are presented for non-normally distributed data.

*dp-ucMGP* dephosphorylated un-carboxylated matrix Gla protein, *PIVKAII* prothrombin induced by vitamin K absence, *hsCRP* high-sensitivity C-reactive protein, *TNF-α* tumor necrosis factor, *IL-6* interleukin 6.

^a^*p* based on a Mann-Whitney *U*-test.

^b^*p* based on a Wilcoxon signed-ranked test.

^c^*p* based on quantile regression adjusted for baseline values and changes of dietary vitamin K intake.

At baseline, serum hsCRP (MD: 0.27; *p*: 0.77), IL-6 (MD: −30.0; *p*: 0.32) and TNF-α (MD: −78.45; *p*: 0.25) were not significantly different between the two groups. After 12 weeks of supplementation with MK-7, the serum levels of hsCRP (MD: −0.59; *p*: 0.02), IL-6 (MD: −43.8; *p*: 0.02) and TNF-α (MD: −65.2; *p*: 0.04) were significantly lower in the treatment group, but the between group differences (after the baseline levels and increases in dietary vitamin K intake were taken into consideration) were not statistically significant (*p* > 0.05) (Table 4).

## Discussion

The current study evaluated the effect of 12 weeks of supplementation with MK-7 on T2DM patients. Body composition indices, including the percentage of body fat, fat mass, fat free mass, muscle mass, bone mass and total body water did not differ significantly between the two groups across the trial. There are very few studies which have evaluated the effect of vitamin K on body composition, but no clinical trials have been reported on diabetic patients. It is important to mention that the osteoblast-specific protein, osteocalcin (OC), has been proposed as the key element in the relationship between fat metabolism and vitamin K intake in humans, since OC has Gla (γ-carboxy-glutamic acid) residues that are formed in a vitamin K-dependent way [25]. In other words, vitamin K supplementation reduces ucOC (uncarboxylated OC) and increases cOC (carboxylated OC) [26]. OC can increase β-cell proliferation, insulin secretion, and sensitivity by stimulating the adiponectin expression of adipocytes [20].

Consistent with our finding, research which supplemented postmenopausal women with 180 mcg/day of MK-7 over a 3 year period found no improvement in body composition, although increased levels of cOC were reported [25]. Nevertheless, for a small subgroup of patients classified as good responders (i.e., above median response to OC carboxylation) a significant decrease in abdominal fat mass and estimated visceral adipose tissue was found [25]. In another study, 60–80 years olds received supplementation with 500 mcg/day of phylloquinone for three years, which lead to a reduction in ucOC but did not change total body fat or lean mass [26]. Furthermore, our findings are also supported by research which found supplementation with 1000 mcg/day of vitamin K1 had no significant effect on the percentage of body fat among postmenopausal women [27].

In contrast to the present study, one animal study found that supplementation with 600 mg/kg of phylloquinone, or 600 mg/kg of MK-4, significantly decreased total fat and visceral fat accumulation in non-diabetic rats, when compared with a control group [28]. Furthermore, a study on mouse bone marrow cell cultures revealed that MK-4, but not phylloquinone, inhibited the formation of adipocytes in bone marrow cells [29]. There are several reasons why the response to vitamin K supplementation would vary between animals and humans. Firstly, research suggests that OC mediates the relationship between vitamin K status and fat metabolism, via adiponectin regulation [28]. However, the form of OC which is effective remains unclear and there are differences in the findings reported, depending on whether they involved animals or humans [30, 31]. Secondly, the difference between animal and human studies could be explained by inter-species differences in the expression of the OC gene and carboxylation. Humans have one gene encoding OC, while mice have three [32, 33]. In mice, circulating OC is often completely carboxylated [33], while in humans it can be carboxylated at 1, 2, or 3 Gla residues [34]. Thus, complete carboxylation or complete uncarboxylation of OC rarely occurs in humans [33, 34].

In the present study, we evaluated the serum levels of dp-ucMGP and PIVKAII, pre and post intervention. MGP in the bloodstream can exist in several forms, including p-cMGP (phosphorylated and carboxylated), p-ucMGP (phosphorylated and un-carboxylated), dp-cMGP (dephosphorylated and carboxylated) and dp-ucMGP (dephosphorylated and un-carboxylated) [35]. However, it is the dp-ucMGP form that is an indicator of systemic changes in vitamin k levels [36, 37], and only this form responds to vitamin K supplementation [36]. Furthermore, MGP is an important marker for the health of blood vessels and there is evidence to suggest that vitamin K supplementation results in reduced vascular calcification and a lower risk of CVD [18, 38]. Furthermore, vitamin K supplementation may increase the carboxylated form of MGP and decrease the uncarboxylated form of MGP, and a low level of dp-ucMGP has been associated with less vascular calcification and CVD risk [18, 38, 39]. Therefore, it appears that vitamin K2 is better than K1 at reducing vascular calcification [19, 40]. Nevertheless, one of the most sensitive markers of vitamin K deficiency in the bloodstream is PIVKAII (also called DCP (Des-gamma-Carboxy Prothrombin) or antagonist II) [41, 42]. PIVKAII is a non-carboxylated form of prothrombin that is derived from the liver [43] and if there is a vitamin K deficiency, its level in the blood will rise to ≥2 ng/mL [42, 44]. In the current research, the serum levels of PIVKAII and ucMGP, as vitamin K markers, significantly decreased in the group following supplementation with MK-7 (*p* < 0.05). However, after adjusting for baseline levels and the increases in vitamin K intake over the study duration, only the between group differences for PIVKAII (*p* < 0.05) remained significant.

In line with our study, research on T2DM patients with CVD found reduced levels of dp-ucMGP in the intervention group, after 3 months of supplementation with 360 mcg/day of MK-7 [21]. In another study, 60 renal transplant recipients were supplemented with 360 mcg/day of MK-7 for 8 weeks. After the intervention period, their dp-ucMGP levels had decreased by a statistically significant 55.1% [45]. Furthermore, research on patients aged 60-80 years old found a significant reduction in dp-ucMGP, following supplementation with 360 mcg/day of K1 over a 3-year period [37].

The effectiveness of lower doses of MK-7 on dp-ucMGP has also been investigated. Fulton et al. [46] reported a reduction of dp-ucMGP in participants aged ≤70 years old who had a history of CVD, after 6 months of supplementation with 100 mcg/day of MK-7. The long-term effects of MK-7 on dp-ucMGP have also been studied by Knapen et al. [47], in which postmenopausal women were supplemented with 180 mcg/day of MK-7 over a three year period. After the intervention period, dp-ucMGP was reduced by 50%, compared with the placebo group.

Previous research has suggested that the effect of MK-7 on dp-ucMGP and PIVKAII is both dose and time dependent [41]. In a 12-week clinical trial involving people aged 40 to 65 years old, the effect of different doses of MK-7 supplementation were found to lead to statistically significant and dose-dependent reductions in dp-ucMGP level. More specifically, the dp-ucMGP level decreased by 31% in the 180 mcg/day group, 46% in the 360 mcg/day group, but remained unchanged in the placebo group [48]. In another study, hemodialysis patients were given different doses of MK-7 (45 mcg/day, 135 mcg/day, and 360 mcg/day) across a 6-week study period. They found a statistically significant and dose-dependent decrease in PIVKAII and dp-ucMGP for the MK-7 groups, in comparison to the control group [41]. Therefore, it seems that if we had extended the duration of our study, or had increased the dosage of MK-7, we may also have seen between group differences in dp-ucMGP.

The present study was the first to investigate the effect of vitamin K on TNF-α and hsCRP in diabetic patients. After 12 weeks of supplementation with MK-7, the serum levels of the inflammatory markers, including hsCRP, IL-6 and TNF-α, were significantly lower in the MK-7 group, but the between groups differences were not significant (after adjusting for baseline levels and the increased intake of dietary vitamin K at the end of the study).

There is very little previous research which has examined the effect of vitamin K supplementation on oxidative stress and inflammation in diabetes [20]. Consistent with the present study, Varsha et al. [49–51] reported a beneficial effect of vitamin K1 supplements on diabetic rats. After giving diabetic rats 5 mg/kg of vitamin K1 for 12 weeks, significant improvements were found in the inflammatory markers they measured. Dihingia et al. [52, 53] also examined the effect of several dose levels of vitamin K1 (1, 3, and 5 mcg/kg/day) over an 8-week period and found a significant dose-dependent reduction in IL-6 among diabetic mice. In addition, further support can be drawn from cross-sectional studies, which have found dietary intake of vitamin K to be negatively related to levels of IL-6‌, CRP‌ and TNF-α [54–56].

In contrast, three studies with non-diabetic patients have reported results, which are inconsistent with our findings [46, 56, 57]. In a study of older men and women, Shea et al. reported that supplementation with 500 mcg/day of vitamin K1 did not lead to improvements in IL-6‌ or CRP [56]. Furthermore, Kristensen et al. [57] conducted a similar clinical trial on postmenopausal women, supplementing them with 500 mcg/day of vitamin K1 over a 6-week period. However, there were no significant improvements in the IL-6 or CRP levels, found following completion of the intervention. In addition, Fulton et al. [46] performed a similar study on people under the age of 70 with a history of vascular disease. After 6 months of supplementation with 100 mcg/day of MK-7, no significant effect on CRP was observed. There are two obvious reasons for the inconsistencies in these findings with those of the present study. Firstly, two of the studies tested low supplementary doses of vitamin K [56, 57] and one of the studies used a short intervention period [57].

The effectiveness of vitamin K supplements for reducing oxidative stress and inflammation are likely due to the fact that vitamin K: (a) suppresses hyperglycemia [49, 50, 52, 58, 59]; (b) increases SOD (superoxide dismutase) and GSH (reduced glutathione), but reduces ROS (reactive oxygen species) via its antioxidant potential [49, 50]; (c) suppresses NF-Kß (nuclear factor kappa ß) production [49, 52]; and (d) reduces AGE (advanced glycation end products) formation [49, 50].

The present study has some strengths and limitations. Firstly, previous research has reported the effects of vitamin K supplementation on animals with DM, but only one clinical trial investigated the effect of MK-7 on T2DM patients, but they were only investigating vascular calcification. Thus, the current research was the first to investigate the beneficial effect of MK-7 on PIVKAII, dp-ucMGP, inflammatory markers and body composition indices among T2DM patients, but more studies are needed to confirm our findings. Secondly, unfortunately we do not know how strictly participants adhered to the nutritional recommendations we provided. Thirdly, it would have been better to measure ucOC and cOC, in order to clarify the probable link between body composition and vitamin K supplementation. Fourthly, it would have been better to use weighted food records (WFR), which are considered to be more precise, instead of 3-day food records to evaluate patient’s dietary intake.

In conclusion, the supplementation of T2DM patients with MK-7 led to improvements in serum levels of PIVKAII. However, the effectiveness of MK-7 supplementation for improving dp-ucMGP levels, inflammatory status (IL-6, TNF- α, and hsCRP) and body composition indices require further research, as there were no statistically significant differences between the two groups. Therefore, MK-7 may be a potentially useful adjunctive treatment for T2DM patients, if the results of the present study are confirmed in future research.

## Supplementary information

Appendix Table 1

## Data Availability

The data are available from the corresponding author, upon reasonable request.
