# Supplementary material for: The effect of menaquinone-7 supplementation on dp-ucMGP, PIVKAII, inflammatory markers, and body composition in type 2 diabetes patients: a randomized clinical trial
Source: Nutr Diabetes. 2022 Apr 1;12:15. doi: 10.1038/s41387-022-00192-5 (PMC8976086; doi:10.1038/s41387-022-00192-5)
Supplement: Supplementary file 1 — Appendix Table 1 [file 41387_2022_192_MOESM1_ESM.docx]

| **Appendix Table 1: dietary recommendations given to the patients at the beginning of the study** |
| --- |
| 1. The patients were advised to start the day with breakfast and that they should not consume food in the late evening. 2. They were reminded to eat low-carbohydrate diets, which may prevent postprandial glucose excursion in type 2 diabetes patients. 3. The importance of consuming a low glycemic diet was strongly emphasised. For example, they should eat brown rice rather than white rice and low-carbohydrate bread rather than normal bread. 4. They were asked to increase their consumption of dietary fiber, which ameliorates postprandial glucose elevation by reducing glucose absorption from the intestines. 5. They were advised to eat a protein rich diet. 6. They were reminded to eat vegetables with a lot of fiber and meat or fish containing a large amount of protein first, and after a short pause to eat the carbohydrates of their meal, including the staple foods. |
